# Lymphopenia predicted illness severity and recovery in patients with COVID-19: A single-center, retrospective study

**DOI:** 10.1371/journal.pone.0241659

**Published:** 2020-11-18

**Authors:** Jiheng Liu, Heng Li, Ming Luo, Jiyang Liu, Lingzhen Wu, Xianfeng Lin, Ruijuan Li, Zhihua Wang, Haiying Zhong, Wenli Zheng, Yan Zhou, Dixuan Jiang, Xin Tan, Zhiguo Zhou, Hongling Peng, Guangsen Zhang

**Affiliations:** 1 Department of Hematology and Oncology, The First Hospital of Changsha, Changsha, Hunan, China; 2 Department of Hematology, The Second Xiangya Hospital, Central South University, Changsha, Hunan, China; 3 Institute of Molecular Hematology, Central South University, Changsha, Hunan, China; 4 Hunan Key Laboratory of Tumor Models and Individualized Medicine, Changsha, Hunan, China; 5 Department of General Surgery, The Second Xiangya Hospital, Central South University, Changsha, Hunan, China; 6 The First Hospital of Changsha, Changsha, Hunan, China; 7 Department of Emergency, The First Hospital of Changsha, Changsha, Hunan, China; 8 Department of Respiratory Medicine, The First Hospital of Changsha, Changsha, Hunan, China; 9 Department of Pediatric, The First Hospital of Changsha, Changsha, Hunan, China; BronxCare Health System, Affiliated with Icahn School of Medicine at Mount Sinai, NY, USA, UNITED STATES

## Abstract

The outbreak of SARS-CoV-2 began in December 2019 and rapidly became a pandemic. The present study investigated the significance of lymphopenia on disease severity. A total of 115 patients with confirmed COVID-19 from a tertiary hospital in Changsha, China, were enrolled. Clinical, laboratory, treatment and outcome data were gathered and compared between patients with and without lymphopenia. The median age was 42 years (1–75). Fifty-four patients (47.0%) of the 115 patients had lymphopenia on admission. More patients in the lymphopenia group had hypertension (30.8% vs. 10.0%, P = 0.006) and coronary heart disease (3.6% vs. 0%, P = 0.029) than in the nonlymphopenia group, and more patients with leukopenia (48.1% vs 14.8%, P<0.001) and eosinopenia (92.6% vs 54.1%, P<0.001) were observed. Lymphopenia was also correlated with severity grades of pneumonia (P<0.001) and C-reactive protein (CRP) level (P = 0.0014). Lymphopenia was associated with a prolonged duration of hospitalization (17.0 days vs. 14.0 days, P = 0.002). Lymphocyte recovery appeared the earliest, prior to CRP and chest radiographs, in severe cases, which suggests its predictive value for disease improvement. Our results demonstrated the clinical significance of lymphopenia for predicting the severity of and recovery from COVID-19, which emphasizes the need to dynamically monitor lymphocyte count.

## Introduction

Coronaviruses (CoV) are a large family of viruses that cause illness ranging from the common cold to more severe diseases, such as Middle East Respiratory Syndrome (MERS-CoV) [1] and Severe Acute Respiratory Syndrome (SARS-CoV) [2]. A novel coronavirus (SARS-CoV-2) was recently detected in Wuhan, China, and it caused a pandemic spread of coronavirus disease (COVID-19) worldwide. Controlling the spread of COVID-19, achieving early diagnosis of this disease and treating it effectively remain highly challenging.

SARS-CoV-2 is a highly infectious zoonotic coronavirus that is 96% identical at the whole-genome level to a bat coronavirus [3]. Common signs of infection include respiratory symptoms, fever, dry cough, and breathing difficulties. Infection causes pneumonia, severe acute respiratory syndrome, septic shock, multiorgan failure and even death in more severe cases [4, 5]. Although COVID-19 shares much in common with SARS and MERS, its epidemiological and clinical features are not fully known.

Lymphopenia and eosinopenia are prominent laboratory abnormalities reported in patients with SARS, and these conditions are more frequent in patients with severe disease compared to nonsevere disease [6]. Lymphopenia is also observed in approximately 60% of patients with SARS-CoV-2 infection at initial presentation [4]. However, the clinical significance and underlying mechanisms of this phenomenon have not been clucidated.

The present study retrospectively analyzed the frequency and association of lymphopenia with COVID-19 severity.

## Materials and methods

### Study design and participants

For this single-centred, retrospective study, a total of 115 patients diagnosed with COVID-19 from the First Hospital of Changsha were included. All patients were admitted from January 17, 2020, to February 14, 2020. Written consent was obtained from all patients, and the Ethical Committee of the First Hospital of Changsha approved the study. The diagnosis was based on clinical criteria and laboratory features according to WHO interim guidance [7]. The final date of follow-up was March 13, 2020.

### Data collection

Epidemiological and clinical data were collected from COVID-19 patients upon hospitalization. Illness severity was defined according to the Chinese management guidelines for COVID-19 (version 7.0) [8]. Mild-grade disease was defined as patients with mild clinical manifestations and no sign of pneumonia on imaging tests. Severe-grade disease was defined as cases that met any of the following criteria: a. respiratory rate exceeded 30 times per minute; b. blood oxygen saturation less than 93%; and c. oxygenation index (PaO_2_/FiO_2_ [pressure of oxygen in arterial blood/fraction of inspire oxygen]) less than 300. Any patients who needed mechanical ventilation because of respiratory failure, who presented with shock, or who were monitored in the intensive care unit (ICU) because of multiple organ failure were deemed critical cases. The other cases were classified as general grade. Blood counts, blood biochemistry, chest radiographs and computed tomographic (CT) scans were performed in the initial days after admission. Therapeutic measures and outcome data were collected from the electronic medical network of the First Hospital of Changsha. Two independent doctors reviewed and verifieded all information to ensure accuracy.

### Laboratory procedures

As previously reported [4], throat-swap specimens were taken from the upper respiratory tract, and confirmation experiments for SARS-CoV2 were performed using real-time RT-PCR following the recommendation of the China National Center for Disease Control. A cycle threshold value (Ct value) less than 37 was defined as a positive test, and a Ct value over 40 was defined as a negative record.

### Imaging data

The imaging data was reviewed blindly and independently by two radiologists (5 and 15 years of experience) using a CT score system to evaluate the extent of disease [9]. The presence or absence of nine imaging features were recorded, which were defined in a previous study performed by the radiologist team from our institution [9], including ground-glass opacities, consolidation, mixed gound-glass opacities and consolidation, traction bronchiectasis, bronchial wall thickening, reticulation, subpleural bands, vascular enlargement and lesion distribution.

### Statistical analyses

Continuous variables are presented as the means (SD) for normally distributed data or medians (IQR) for non-normally distributed data. Categorical variables are described as counts (%). Statistical analyses were performed using the Pearson χ2 test, Fischer’s exact test, Mann–Whitney *U*-test, and Kruskal-Wallis *H*-test followed by a post hoc test. Kaplan–Meier methodology and log-rank tests were used to compare the duration of hospitalization and the time to recovery (TTR) in patients with or without lymphopenia. TTR was defined as the length of time from admission to the date when lung images showed signs of improvement. Variables that were significant on univariate analysis were further included on multivariate analysis using Cox’s regression. Analyses were performed using the SPSS statistical package (SPSS, Chicago, IL, USA) and GraphPad Prism (GraphPad Software, San Diego, CA, USA). P-values<0.05 were considered significant.

## Results

### Clinical characteristics of patients

The clinical characteristics are summarized in Table 1, and the patients were divided into two groups depending on the presence of lymphopenia (lymphocyte count <1.0× 10⁹/L). Fifty-four of the enrolled patients (47.0%) had lymphopenia upon admission to the hospital. The median age of all patients was 42 years (IQR 1–75). Four patients (3.5%) were under 14 years old, and three patients had no pneumonia. The median age of patients with lymphopenia on admission was 49 years (IQR 21–75), which was significantly older than patients with no lymphopenia. Most patients (73/93 [78.5%]) had specific exposure history, which was previous travel to Wuhan or exposure to a patient diagnosed with COVID-19. Less than half (38/112 [33.9%]) of the patients had chronic complications, including hypertension (22/112 [19.6%]), diabetes (9/112 [8.0%]), cardiovascular disease (4/112 [3.6%]) and hepatitis B (6/112 [5.4%]). Notably, there were more patients with hypertension (P = 0.006) and coronary heart disease (P = 0.029) in the group of lymphopenic patients than in patients with no lymphopenia. In contrast, all six patients with chronic hepatitis B had no lymphopenia upon admission (P = 0.019).

**Table 1:** Clinical and laboratory characteristics of patients with SARS-CoV2 infection.

	Total (N = 115)	Patients with lymphopenia (n = 54)	Patients with no lymphopenia (n = 61)	*P* value
**General characteristics and clinical manifestation**				
Sex				0.322
Male	61 (53.0%)	26 (48.1%)	35 (57.4%)	
Female	54 (47.0%)	28 (51.9%)	26 (42.6%)	
Age, years (range)	42 (1–75)	49 (21–75)	40 (1–72)	0.001
Exposure history	73/93 (78.5%)	23/37 (62.2%)	50/56 (89.3%)	0.002
Comorbidity	38/112 (33.9%)	21/52 (40.4%)	17/60 (28.3%)	0.179
Hypertension	22/112 (19.6%)	16/52 (30.8%)	6/60 (10.0%)	0.006
Diabetes	9/112 (8.0%)	5/52 (9.6%)	4/60 (6.7%)	0.567
Coronary heart disease	4/112 (3.6%)	4/52 (7.7%)	0/60 (0%)	0.029
COPD	4/112 (3.6%)	3/52 (5.8%)	1/60 (1.7%)	0.243
Hepatitis B	6/112 (5.4%)	0/52 (0%)	6/60 (10.0%)	0.019
Others	8/112 (7.1%)	4/52 (7.7%)	4/60 (6.7%)	0.834
Onset symptoms				
Fever	73/97 (75.3%)	34/37 (91.9%)	39/60 (65.0%)	0.003
Cough	60/97 (61.9%)	26/37 (70.3%)	34/60 (56.7%)	0.18
chill	10/97 (10.3%)	4/37 (10.8%)	6/60 (10.0%)	0.898
Fatigue	44/97 (45.4%)	21/37 (56.8%)	23/60 (38.3%)	0.077
Muscle soreness	10/97 (10.3%)	5/37 (13.5%)	5/60 (8.3%)	0.415
Nausea or vomiting	4/97 (4.1%)	2/37 (5.4%)	2/60 (3.3%)	0.618
Diarrhea	5/97 (5.2%)	3/37 (8.1%)	2/60 (3.3%)	0.302
Time from illness onset to hospital admission, days	5.5 (1–40)	5.0 (1–25)	6.0 (1–40)	0.05
**Laboratory test and imaging data**				
WBC count, × 10⁹/L	4.87 (1.75–17.11)	4.08 (1.75–14.71)	5.72 (2.63–17.11)	0.008
<4.0	35 (30.4%)	26 (48.1%)	9 (14.8%)	<0.001
Lymphocyte count, × 10⁹/L	1.06 (0.17–9.54)	0.76 (0.17–1.82)	1.51 (1.02–9.54)	<0.001
Eosinophil count, × 10⁹/L	0.02 (0.00–0.42)	0.00 (0.00–0.42)	0.05 (0.00–0.35)	0.002
<0.02	83 (72.2%)	50 (92.6%)	33 (54.1%)	<0.001
Hemoglobin, g/L	129 (77–174)	126 (77–168)	133 (89–174)	0.071
Anemia	17 (14.8%)	11 (20.4%)	6 (9.8%)	0.112
Platelet count, × 10⁹/L	181 (35–685)	163 (78–334)	217 (35–685)	0.015
<100				
ALT, U/L	19.70 (5.40–79.37)	20.02 (10.88–55.30)	19.70 (5.40–79.37)	0.795
>40	11/105 (10.5%)	4/44 (9.1%)	7/61 (11.5%)	0.694
Globulin, g/L	25.32 (18.20–34.67)	25.46 (20.38–32.9)	25.20 (18.20–34.67)	0.792
Albumin, g/L	37.86 (27.91–47.35)	36.13 (28.12–44.52)	38.87 (27.91–47.35)	0.001
Creatinine, umol/L	48.29 (5.17–255.71)	48.46 (20.58–255.71)	48.29 (5.17–229.8)	0.773
LDH, U/L	161.5 (88.2–463.8)	187.7 (88.2–463.8)	152.4 (107.1–379.7)	0.022
C-reactive protein	13.07 (0.1–101.94)	24.53 (0.7–91.6)	8.92 (0.1–101.94)	0.005
ESR	35.5 (3.0–552.0)	43.5 (0.3–552.0)	28.5 (4.0–115.0)	0.044
Bilateral lung involvement in lung CT scan	64/92 (69.6%)	26/33 (78.8%)	38/59 (64.4%)	0.15
Disease severity status				<0.001
Mild	13 (11.3%)	2 (3.7%)	11 (18.0%)	
General	74 (64.3%)	30 (55.6%)	44 (72.1%)	
Severe	22 (19.1%)	17 (31.5%)	5 (8.2%)	
Critical	6 (5.2%)	5 (9.3%)	1 (1.6%)	
**Treatment**				
Antiviral treatment				
Lopinavir and ritonavir	78/95 (82.1%)	34/37 (91.9%)	44/58 (75.9%)	0.047
Interferon beta-2b	37/95 (38.9%)	14/37 (37.8%)	23/58 (75.9%)	0.859
Recombinant human cytokine derived protein	48/95 (38.9%)	21/37 (56.8%)	27/58 (46.6%)	0.332
Antibacterial treatment	48/94 (51.1%)	27/36 (75.0%)	21/58 (36.2%)	<0.001
Systemic corticosteroid treatment	41/105 (39.0%)	29/46 (63.0%)	12/59(20.3%)	<0.001
Human γ-immunoglobulin	32/94 (34.0%)	19/36 (52.8%)	13/58 (22.4%)	0.003
Respiratory support				0.013
Nasal cannula	67/91 (73.6%)	22/35 (62.9%)	45/56 (80.4%)	
High-flow nasal cannula	17/91 (18.7%)	9/35 (25.7%)	8/56 (14.3%)	
Non-invasive ventilation	1/91 (1.1%)	1/35 (2.9%)	0/56 (0%)	
Invasive ventilation	3/91 (3.3%)	3/35 (8.6%)	0/56 (0%)	
**Prognosis**				
Improved and discharged	113(98.3%)	52 (96.3%)	61 (100%)	0.317
Inpatient treatment	1 (0.9%)	1 (1.9%)	0 (0%)	
Death	1 (0.9%)	1 (1.9%)	0 (0%)	

Data are median (IQR), n (%), or n/N (%), where N is the total number of patients with available data. P values comparing patients with or without lymphopenia are from a χ^2^ test, Fisher’s exact test, or Mann-Whitney U test. COPD = chronic obstructive pulmonary disease; WBC = white blood cell; ALT = alanine aminotransferase; LDH = lactate dehydrogenase; ESR = erythrocyte sedimentation rate.

The most common onset symptoms were fever (75.3%), dry cough (61.9%), fatigue (45.4%), chill (10.3%) and muscle soreness (10.3%). More patients in the lymphopenia group had fever as the initial symptom (91.9% vs. 65.0%, P = 0.003). The median time from the appearance of symptoms to admission was 5.5 days (IQR 1–40).

More leukopenia (white blood cell count<4.0×10⁹/L), eosinopenia (eosinophil count<0.02×10⁹/L) and thrombocytopenia (platelet count<100× 10⁹/L) were observed in patients with lymphopenia (P = 0.008, 0.002 and 0.015, respectively) than patients with normal lymphocyte counts. The median albumin level was 36.13 g/L (IQR [28.12–44.52]) in patients with lymphopenia, which was significantly less than patients with no lymphopenia (P = 0.001). More patients in the lymphopenia group had elevated lactate dehydrogenase (LDH) levels (187.7 U/L [IQR 88.2–463.8] vs. 152.4 U/L [IQR 107.1–379.7], P = 0.002). Inflammatory indicators, such as C-reactive protein (CRP, median 13.07 mg/L [IQR 88.2–463.8]) and erythrocyte sedimentation rate (median 35.5 mm/h [IQR 3.0–552.0]) were elevated. However, both indicators were even higher in patients with lymphopenia (P = 0.005 and 0.044, respectively).

### Association of lymphopenia with the severity of pneumonia

**Fig 1:**
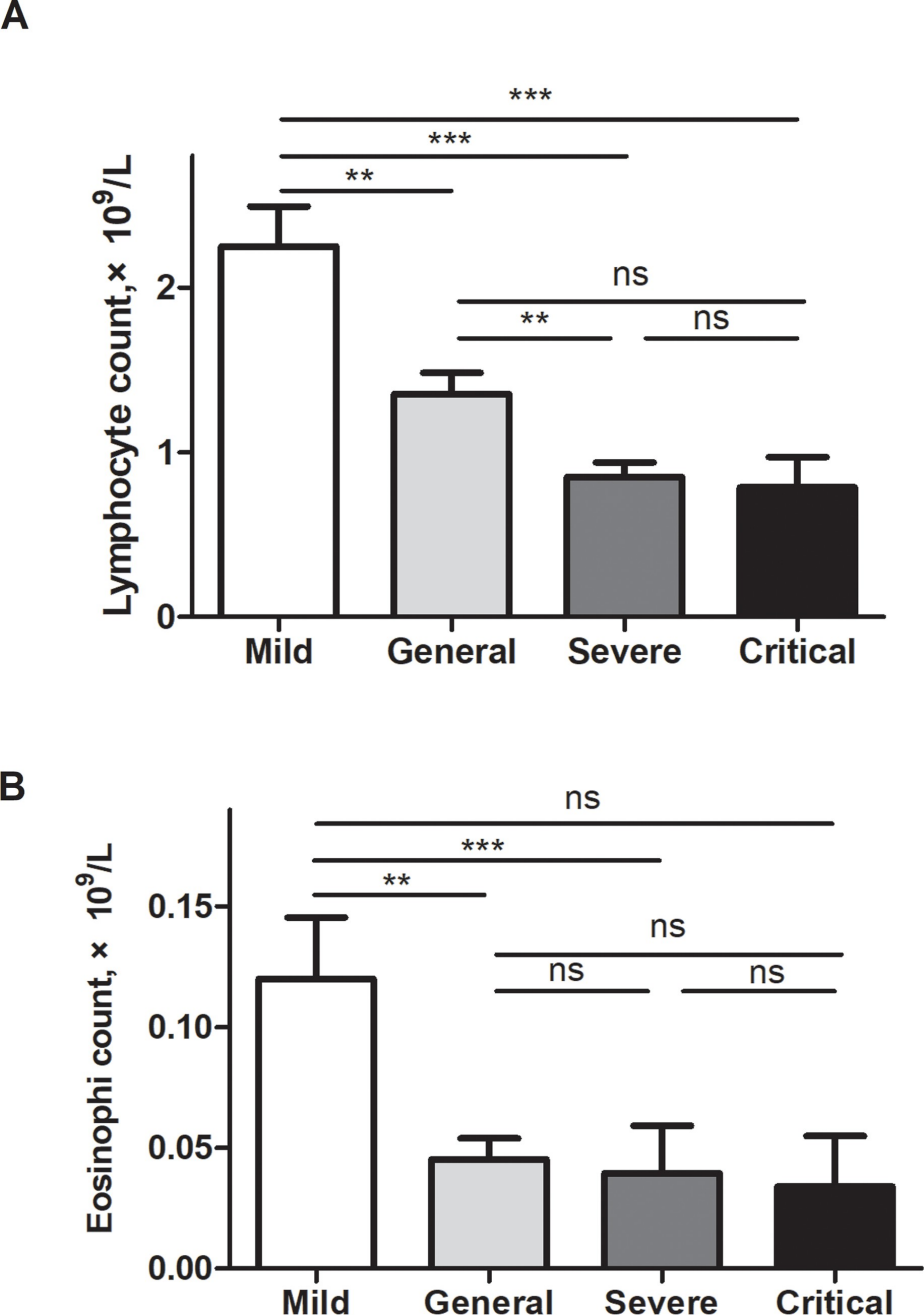
Lymphocyte and eosinophil counts in different groups of illness severity of COVID-19. (A) The median lymphocyte counts were significantly different among the four groups (P<0.0001). The median lymphocyte count of mild cases was 2.45 (0.98–3.81) × 10⁹/L, which was significantly higher than in general cases (1.12 [0.43–9.54] × 10⁹/L, P<0.01), severe cases (0.77 [0.37–2.26] × 10⁹/L, P<0.001) and critical cases (0.74 [0.17–1.56] × 10⁹/L, P<0.001). The median lymphocyte count of general cases was significantly higher than that of severe cases (Fig 1A, P<0.01). We further compared the association of eosinophil counts and disease severity. (B) The median eosinophil counts were significantly different among the four groups (P = 0.0009). The median eosinophil count of mild cases was 0.09 (0.03–0.3) × 10⁹/L, which was significantly higher than that in the general cases (0.01 [0.00–0.35] × 10⁹/L, P<0.01) and severe cases (0.01 [0.00–0.42] × 10⁹/L, P<0.001), but there was no difference between mild cases and critical cases (0.00 [0.00–0.09] × 10⁹/L). ***, P<0.001; **, P<0.01; *, P<0.05.

COVID-19 disease severity was classified into four grades according to the Chinese management guidelines: mild, general, severe and critical. Most of the 115 cases of COVID-19 were classified as general cases (74 [64.3%], Table 1). Only 13 patients (11.3%) were mild cases, i.e., patients with no evidence of pneumonia. The median age in this category was 29 years (IQR 8–67), which was significantly younger than in other categories (P<0.001). Twenty-two patients with lymphopenia (40.8%) were in the severe or critical category, which was significantly greater than patients with no lymphopenia (9.8%, P<0.001). A higher incidence of lymphopenia was observed with more severe illness. A Kruskal-Wallis test was used to compare the lymphocyte count among the four groups of different severity grades. As shown in Fig 1A, the median lymphocyte counts were significantly different among the four groups (P<0.0001). The median lymphocyte count of mild cases was 2.45 (0.98–3.81) × 10⁹/L, which was significantly higher than that of general cases (1.12 [0.43–9.54] × 10⁹/L, P<0.01), severe cases (0.77 [0.37–2.26] × 10⁹/L, P<0.001) and critical cases (0.74 [0.17–1.56] × 10⁹/L, P<0.001). The median lymphocyte count of general cases was significantly higher than that of severe cases (Fig 1A, P<0.01). We further compared the association of eosinophil count and disease severity. As shown in Fig 1B, the median eosinophil counts were significantly different among the four groups (P = 0.0009). The median eosinophil count of mild cases was 0.09 (0.03–0.3) × 10⁹/L, which was significantly higher than that in the general cases (0.01 [0.00–0.35] × 10⁹/L, P<0.01) and severe cases (0.01 [0.00–0.42] × 10⁹/L, P<0.001), but there was no difference between the mild and critical cases (0.00 [0.00–0.09] × 10⁹/L).

**Fig 2:**
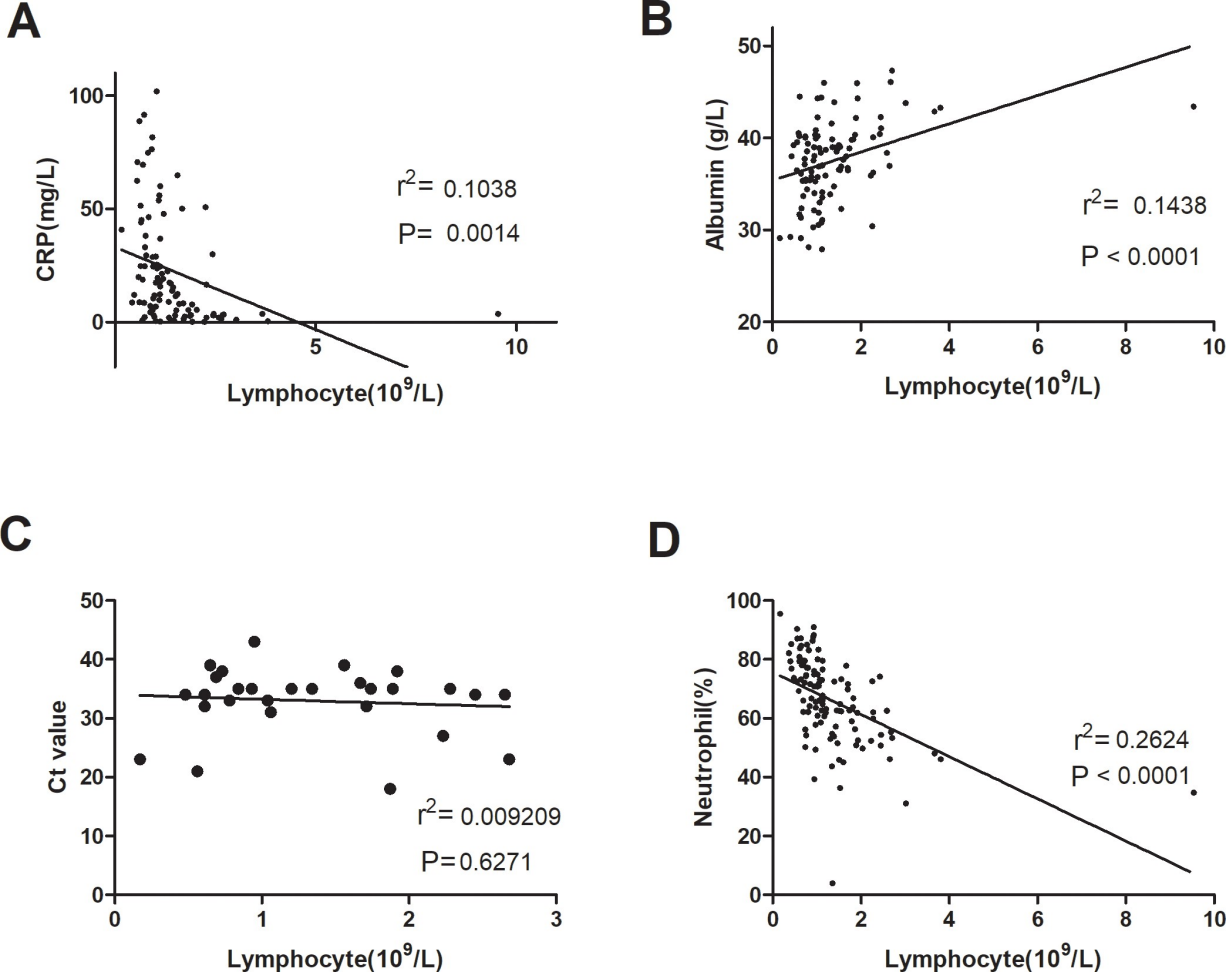
Correlation of lymphocyte count and laboratory tests. Lymphopenia was highly correlated with CRP levels (A), serum albumin levels (B) and neutrophil counts (D). No significant correlation was found between lymphocyte count and Ct value (C). Spearman rank correlation analysis (r) and P values are provided in each graph. CRP, C-reactive protein; Ct value, cycle threshold value.

Lymphopenia was highly correlated with laboratory manifestations in SARS-CoV-2-infected patients (Fig 2). Lymphocyte counts were inversely related to CRP levels (Fig 2A, P = 0.0014) and neutrophil counts (Fig 2D, P<0.0001) and were positively associated with serum albumin levels (Fig 2B, P<0.0001). However, there was no significant correlation between lymphocyte counts and Ct values (Fig 2C).

### Predictive value of lymphopenia for disease severity was comparable with CRP

**Fig 3:**
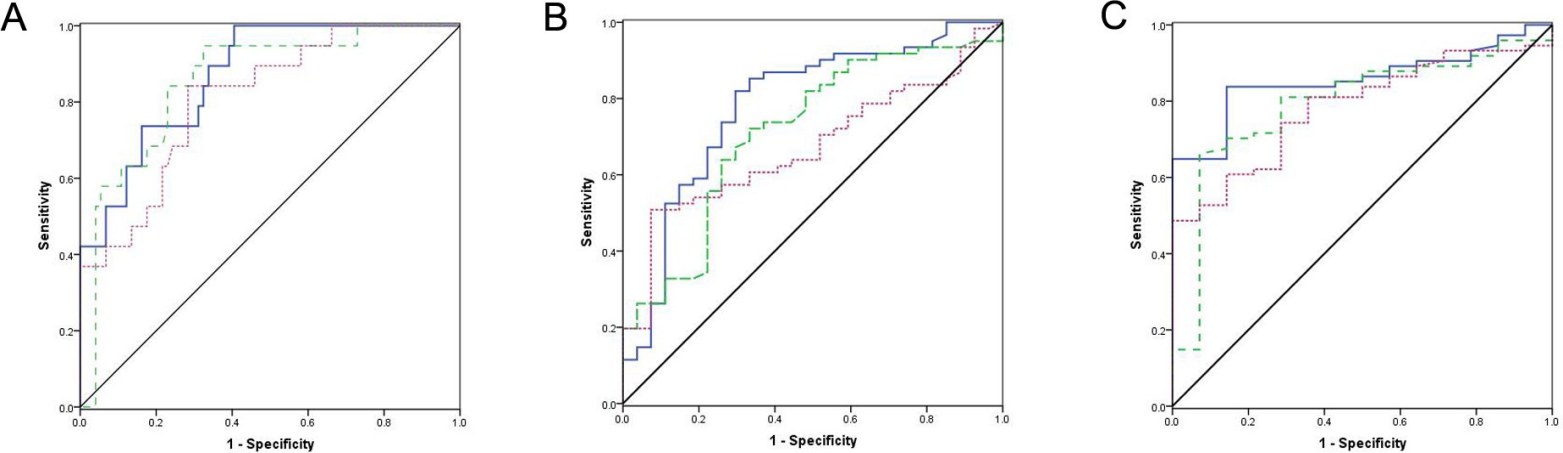
ROC plots express the prognostic value of illness severity of lymphopenia compared with CRP and LDH levels. ROC curves to predict patients with (A) pneumonia of severe grade or critical grade, (B) bilateral lung involvement in lung CT scan, and (C) abnormal lung image on discharge. The diagonal line indicates an AUC of 0.5 (no discrimination between the two states). The blue line indicates CRP level; The green dashed line indicates lymphocyte count; The pink dotted line indicates LDH level. LYM, lymphopenia; CRP, C-reactive protein; LDH, lactate dehydrogenase; AUC, area under the curve; ROC, receiver-operator curve.

CRP and LDH levels are used in the management of infection. Therefore, we next analyzed the value of lymphopenia for assessing the severity of COVID-19 compared with these inflammatory markers. Receiver-operator curve (ROC) plots were used to express the prognostic value of these parameters for illness severity based on grades of pneumonia, bilateral lung involvement in lung CT scan and the presence of abnormal lung image on discharge (see Fig 3). For the prediction of severe or critical disease, the area under the curve (AUC) of lymphopenia was 0.854 compared to 0.870 for CRP and 0.810 for LDH (Fig 3A). For the prediction of bilateral lung involvement, the AUC of lymphopenia was 0.714 compared to 0.782 and 0.672 for CRP and LDH, respectively (Fig 3B). For the prediction of abnormal lung images on discharge, the AUC of lymphopenia was 0.792 compared to 0.856 and 0.782 for CRP and LDH, respectively (Fig 3C).

To investigate the predictive value of all three variables combined, we further created a scoring system by assigning 1 point for lymphopenia, elevated CRP level and elevated LDH level. ROC plots were used to express the prognostic value of the scoring system for disease severity, as shown in S1 Fig. For the prediction of severe or critical disease, the AUC was 0.738 (S1A Fig). For the prediction of bilateral lung involvement, the AUC was 0.714 (S1B Fig). For the prediction of abnormal lung image on discharge, the AUC was 0.827 (S1C Fig).

### Lymphopenia was a risk factor for prolonged hospitalization

**Fig 4:**
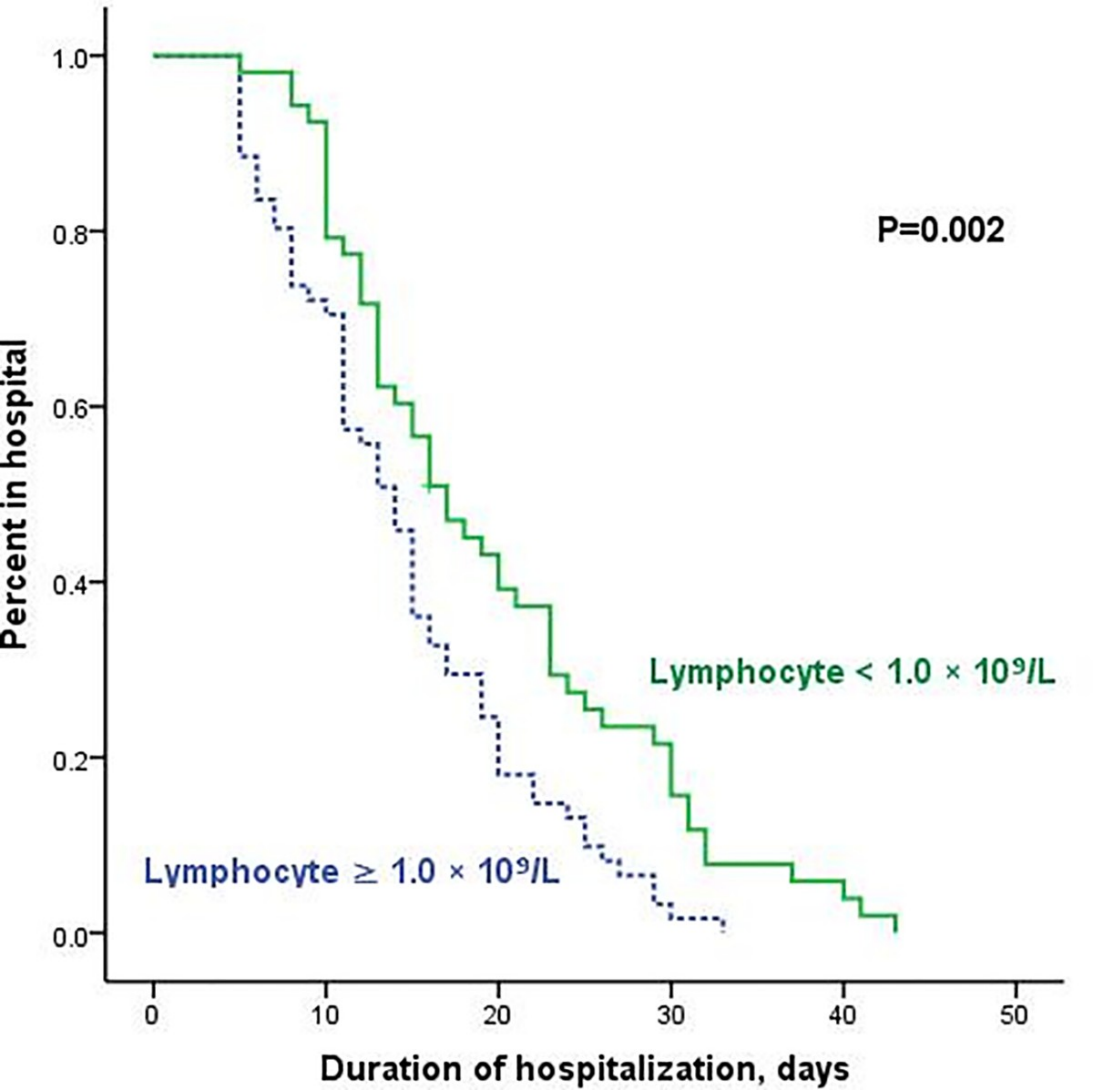
Kaplan-Meier curves for the duration of hospitalization according to lymphocyte count.

Risk factors for the duration of hospitalization were analyzed. As shown in Fig 4, lymphopenia was an indicator for prolonged hospitalization. The median duration of hospitalization for patients with lymphopenia was 17.0 days (95% confidence interval [CI] 13.007–20.993), which was significantly longer than for patients with no lymphopenia (median 14.0 days [95% CI 12.093–15.907]; P = 0.002).

For the severity grades of COVID-19, the presentation of respiratory failure and requirement of ICU care were also associated with the duration of hospitalization (P<0.001 and P<0.001, respectively; S2 Fig). However, eosinopenia, elevated CRP and comorbidities had no impact on the duration of hospitalization (S2D–S2F Fig). Multivariate analysis was further performed using Cox’s regression. Only respiratory failure was identified as an independent risk factor for the prolonged duration of hospitalization (S1 Table).

**Fig 5:**
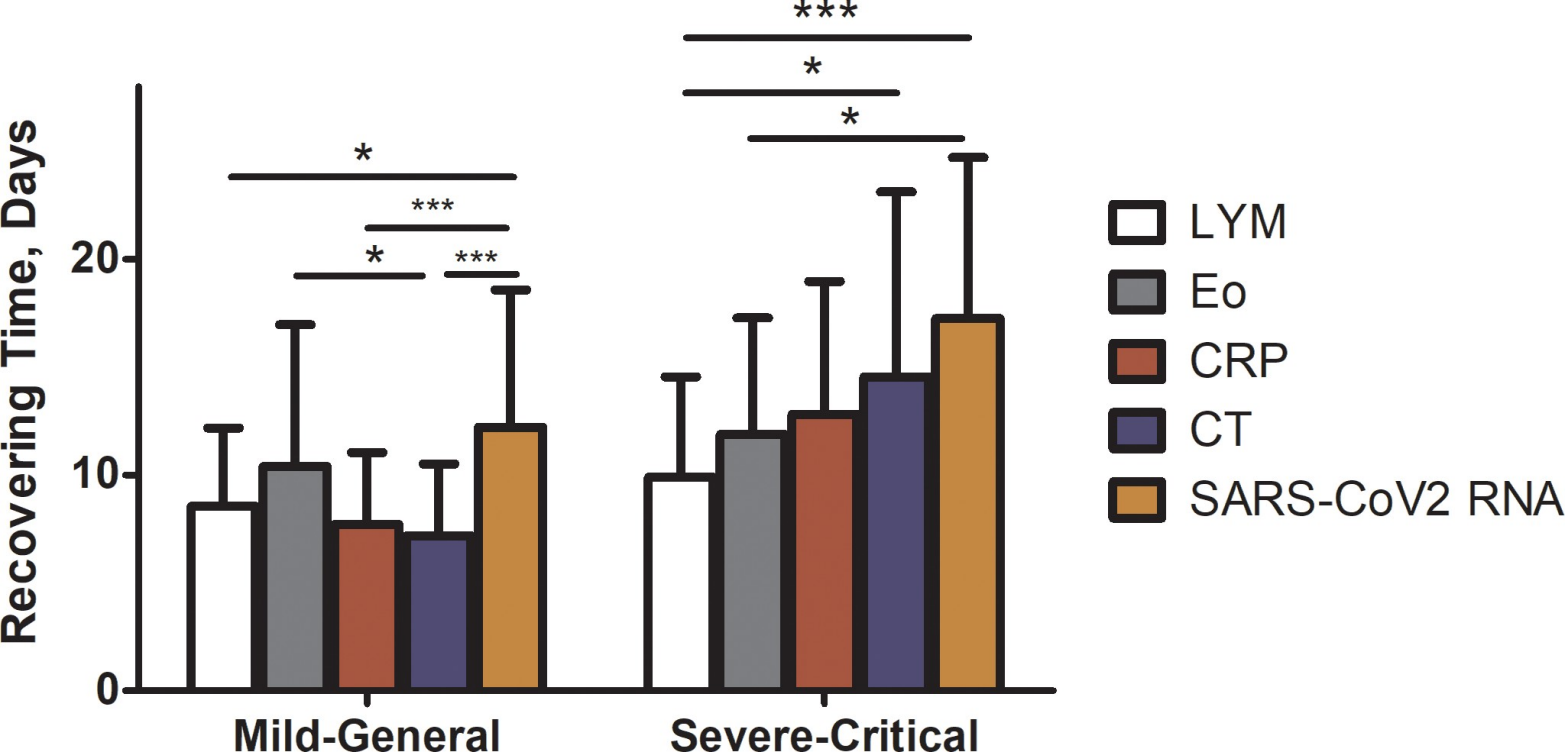
Recovery time (from admission to the date of normalization) of lymphopenia, eosinopenia, CRP level, chest radiograph and clearance time of SARS-CoV2 RNA (from admission to the date of second negative detection result of SARS-CoV2 RNA) was compared using a two-way ANOVA. The recovery time of those indicators above were significantly different (P = 0.0001). In mild and general cases, the median recovery time of lymphocyte was 9.0 (3.0–14.0) days, which was significantly shorter than that of SARS-CoV2-RNA (median 11.0 [3.0–32.0] days, P<0.05); the median recovery time of CRP was 7.5 (1.0–14.0) days, which was significantly shorter than that of SARS-CoV2-RNA (P<0.001); the median recovery time of CT scan was 7.0 (2.0–16.0) days, which was significantly shorter than that of eosinophils (median 9.0 [2.0–26.0] days, P<0.05) and SARS-CoV2-RNA (P<0.001). In severe and critical cases, the median recovery time of lymphocyte was 9.0 (3.0–23.0) days, which was significantly shorter than recovery as evidenced by CT scan (median 12.5 [4.0–32.0] days, P<0.05) and SARS-CoV2-RNA (median 15.0 [8.0–31.0] days, P<0.001). The median recovery time of eosinophils was 12.0 (3.0–23.0) days, which was significantly shorter than that of SARS-CoV2-RNA (P<0.05). The comparisons between other indicators were not significant. LYM, lymphopenia; Eo, eosinopenia; CRP, C-reactive protein. ***, P<0.001; **, P<0.01; *, P<0.05.

We further compared the recovery time of lymphocyte, eosinophil, CRP levels and chest radiographs before the clearance of SARS-CoV2 RNA. For monitoring the status of pneumonia, CT scans were obtained every 3–5 days. No significant difference was found regarding the interval of imaging between lymphopenic and normocytic groups. Because many patients still presented abnormal lung images at discharge, the recovery time of chest radiographs was defined as the length of time from admission to the date when lung images showed signs of improvement. Notably, among the 71 patients who presented with an elevated CRP level, only 45 (63.4%) had a normalized CRP before discharge. Among the 80 patients whose chest radiographs showed lung lesions, only 6 patients’ CT scans were normal on discharge. Six patients (7.5%) still showed no sign of improvement on CT scans before discharge despite a recovery of symptoms and clearance of SARS-CoV2 RNA. Compared with CT scan (median recovery time 12.5 days [4.0–32.0]) and CRP levels (median recovery time 12.0 days [4.0–23.0]), the recovery of lymphocytes was significantly faster (median recovery time 9.0 days [3.0–23.0], see Fig 5) in severe and critical cases, which indicated that normalizing of lymphocyte counts could predict disease improvement.

### Patients with lymphopenia did not benefit from human γ-immunoglobulin treatment

Treatment records were available for 95 of the 115 patients, and these data are summarized in Table 1. More patients in the lymphopenia group required higher levels of respiratory support, such as high-flow nasal cannula (25.7% vs. 14.3%), noninvasive ventilation (2.9% vs. 0%) and invasive ventilation (8.6% vs. 0%, P = 0.013). All 95 patients received antiviral treatment. The most commonly used drugs were lopinavir and ritonavir (82.1%), which were more frequently used in patients with lymphopenia (91.9% vs. 75.9%, P = 0.047). Antibacterial treatment was used in 48 (51.1%) patients. More patients received antibiotics in the lymphopenia group than in the nonlymphopenia group (75.0% vs. 36.2%, P<0.001). Also, 39.0% of patients received 40–80 mg methylprednisolone per day for median 8 (1–18) days. In 63.0% of the patients with lymphopenia, methylprednisolone was used, which was significantly more than the patients with no lymphopenia (20.3%, P<0.001). The duration of methylprednisolone was compared between lymphopenic group and normocytic group, and no significant difference was found between both groups (median 8 days vs. 9 days, P = 0.627). Human γ-immunoglobulin (IVIG) was used in about half of the patients with lymphopenia (52.8%), which was also significantly more than patients with no lymphopenia (22.4%, P<0.001).

**Fig 6:**
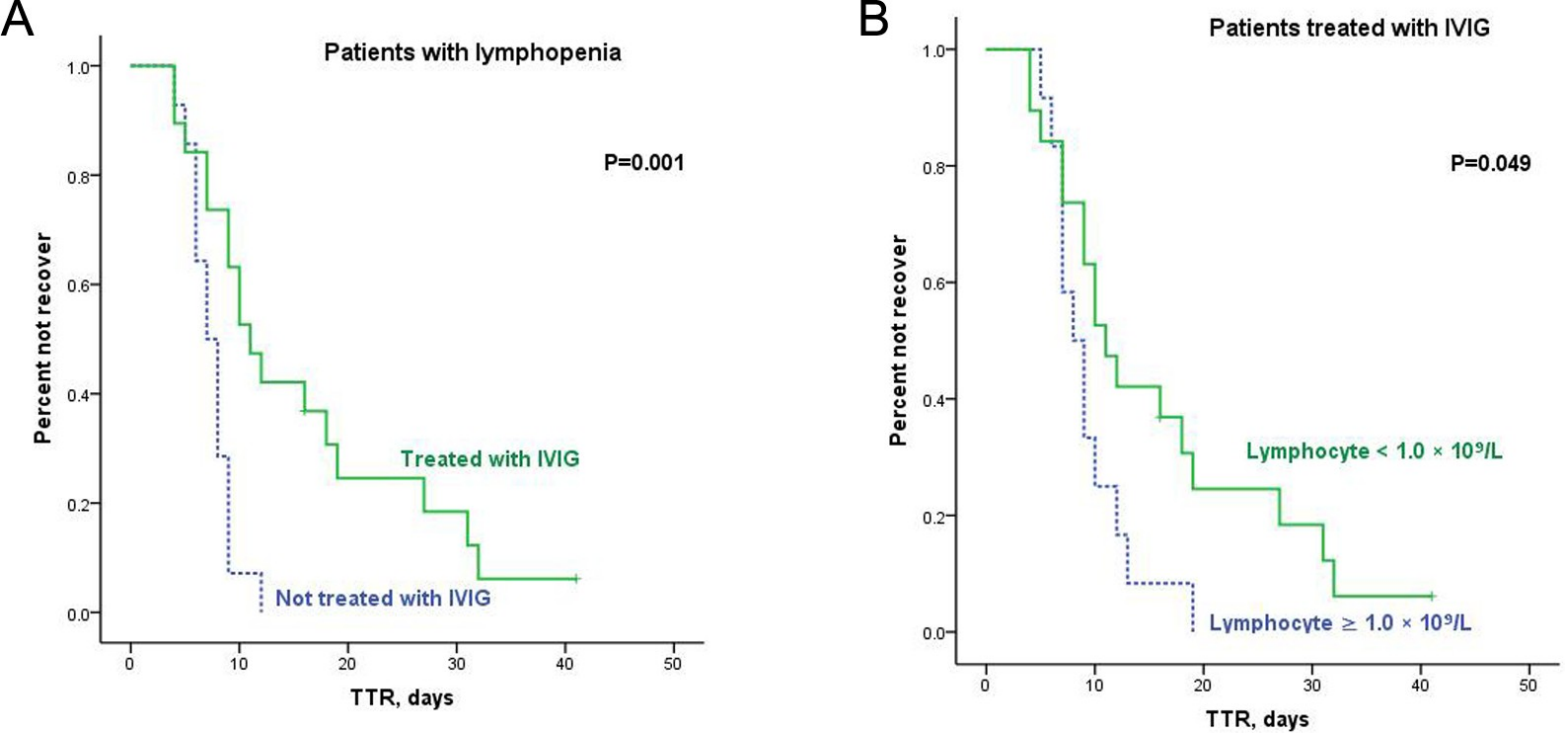
Kaplan-Meier curves for TTR in patients receiving IVIG treatment. (A) Kaplan-Meier curves for TTR according to treatment of IVIG in patients with lymphopenia; (B) Kaplan-Meier curves for TTR according to lymphocyte count in patients treated with IVIG.

To investigate whether patients benefitted from IVIG or corticosteroid treatment, Kaplan-Meier curves were constructed based on therapeutic choice and lymphocyte count (Fig 6 and S3 Fig). The median TTR of patients with lymphopenia treated with IVIG was 11.0 days (95% CI 7.801–14.199), which was significantly longer than patients without IVIG treatment (median 7.0 days [95% CI 5.533–8.467], P = 0.001, Fig 6A). Therefore, we compared the patient composition and found more severe or critical cases in the IVIG group than patients without IVIG treatment (73.7% vs. 0%, P<0.001). Lymphopenia remained an adverse factor on TTR for all patients who received IVIG treatment (median TTR 8.0 days [95% CI 5.737–10.263] vs. 11.0 days [95% CI 7.801–14.199], P = 0.049, Fig 6B).

IVIG treatment in patients without lymphopenia had no benefit (P = 0.190, S3A Fig). No significant benefit from corticosteroid treatment was observed in patients with (S3B Fig) or without lymphopenia (S3C Fig). There was no difference in TTR between patients who received corticosteroid treatment with and without lymphopenia (P = 0.201, S3D Fig).

## Discussion

The present study retrospectively analyzed the characteristics of patients infected with SARS-CoV-2 and identified that lymphopenia was a good predictor for the severity and recovery of COVID-19. Particularly, lymphopenia was associated with inflammatory markers, grades of pneumonia severity and prolonged hospitalization. Normalization of lymphocyte count indicated recovery of COVID-19.

Approximately one third of the COVID-19 patients had a chronic underlying disease, and severe or critical cases accounted for approximately one fourth, which is less than that of other cohorts reported in Wuhan [4, 5, 10]. Notably, more patients with lymphopenia had hypertension and coronary heart disease (Table 1). The underlying reason may be an imbalance of the angiotensin-converting enzyme (ACE)/ACE2 axis. ACE2 is the receptor for SARS-CoV [11] and SARS-CoV2 [12], and it is a surface molecule localized on arterial and venous endothelial cells, arterial smooth muscle cells and cells of the respiratory tract [13]. ACE inhibitors (ACEIs) and angiotensin II receptor blockers (ARBs) decrease the level of ACE and increase the level of ACE2 [14], which may increase the risk of SARS-CoV-2 infection, which may be the reason why more patients with hypertension and coronary heart disease also had lymphopenia, as ACEIs and ARBs are commonly used drugs under these circumstances.

The characteristics and pathogenesis of COVID-19 are similar to the prior severe acute respiratory syndrome (SARS) and Middle East respiratory syndrome (MERS) [2, 4, 15]. However, the clinical manifestation of COVID-19 may be more asymptomatic. Approximately 25% in our patient cohort had no fever on admission (Table 1), which suggests that it is more challenging to identify COVID-19 patients and control the pandemic worldwide.

Risk factors for adverse outcomes in patients with SARS and MERS were investigated in previous studies [1, 15–18]. Lymphopenia is common in SARS and MERS patients, and it is an important predictor for severe disease in SARS [19] and MERS [20]. Approximately half of the patients in the current study presented with lymphopenia on admission, which is comparable with the frequency in SARS [19], but it was lower than the frequency of lymphopenia reported in COVID-19 patients in Hubei Province [21]. Our results revealed that lymphopenia was associated with disease severity, which is consistent with the study by Liu Y et al [22], who found that lymphopenia positively correlated with the severity of acute lung injury in patients with COVID-19. As shown in Fig 5, the recovery of lymphocyte count was the first sign to appear in severe and critical cases before the patients improved and were discharged, which suggests that normalization of lymphocytes was a more sensitive indicator than CRP or CT scan for the prediction of disease recovery in patients of severe and critical grades.

Lymphopenia was reported in varies types of virus-infected diseases, such as SARS [6, 19, 23], MERS [17, 20] and respiratory syncytial virus [24]. A previous study reported that lymphopenia in SARS may be due to enhanced vascular sequestration associated with increased soluble vascular cell adhesion molecule-1 levels [25], but the mechanism is not clear in patients with COVID-19. Glucocorticoid treatment results in lymphopenia because it induces the migration of lymphocytes from the peripheral blood [26]. Viral infections inevitably lead to activation of the hypothalamic-pituitary-adrenal axis under stress, which results in the upregulation of endogenous corticosteroids [27], which may be involved in the immunopathogenesis of lymphopenia of COVID-19. In this study, patients treated with methylprednisolone had a longer recovery time of lymphocyte than patients without methylprednisolone treatment (S2 Table). This result was observed partly because patients received methylprednisolone because of a more aggressive clinical course. On the other hand, the use of corticosteroids could have an impact on lymphocyte counts. However, the recovery time of lymphocyte counts was still the most sensitive predictor for disease recovery.

No specific antiviral treatment is available for SARS, MERS or COVID-19. A range of treatments, including lopinavir/ritonavir, interferon-β2b and recombinant human cytokine-derived protein, were used in this cohort of patients, but no improvements in outcome were observed. IVIG was used as a previous therapeutic measure for SARS, but it was not confirmed as effective for outcome improvement [28]. Our study found no benefit from IVIG in patients with or without lymphopenia. Given the high cost and subsequent economic burden of IVIG on public health systems, more investigations of this approach are needed to provide evidence for a large-scale use.

Our study has several limitations. For example, some of the laboratory examination records and treatment records were not available because this study was retrospective. An examination of lymphocyte subsets was unavailable in most patients, and there is still no evidence regarding which subset contributed to the profound lymphopenia in COVID-19 patients. Therefore, a more comprehensive and thorough investigation is necessary in the future.

In summary, analyses of the clinical data of 115 patients with COVID-19 showed that lymphopenia and eosinopenia were common and correlated with the severity of COVID-19. To the best of our knowledge, this study is the first retrospective study demonstrating the significance of the recovery of lymphocyte count for predicting disease improvement, which emphasizes the need to dynamically monitor blood cell counts in the management of COVID-19.

## Supporting information

S1 Fig ROC plots express the prognostic value of illness severity using a scoring system that combined lymphopenia, CRP and LDH level. ROC curves to predict patients with (A) pneumonia of severe grade or critical grade, (B) bilateral lung involvement in lung CT scan, (C) abnormal lung image on discharge. The diagonal line indicates an AUC of 0.5 (no discrimination between the two states). CRP, C-reactive protein; LDH, lactate dehydrogenase; AUC, area under the curve; ROC, receiver-operator curve.
(TIF)

S2 Fig Kaplan-Meier curves for the duration of hospitalization in patients within different categories. (A) Kaplan-Meier curves for duration of hospitalization according to severity grades of COVID-19; (B) Kaplan-Meier curves for duration of hospitalization according to the presentation of respiratory failure; (C) Kaplan-Meier curves for duration of hospitalization according to the requirement of ICU care; (D) Kaplan-Meier curves for duration of hospitalization according to eosinophil count. The blue line indicates patients with eosinophil counts ≥0.02 × 10⁹/L; The green line indicates patients with eosinophil counts <0.02 × 10⁹/L; P = 0.793; (E) Kaplan-Meier curves for duration of hospitalization according to the CRP levels. The blue line indicates patients with a normal CRP level; The green line indicates patients with an elevated CRP level; P = 0.094. (F) Kaplan-Meier curves for duration of hospitalization according to comorbidity. The blue line indicates patients with no comorbidity; The green line indicates patients with comorbidities; P = 0.782. CRP, C-reactive protein. RF, respiratory failure; ICU, intensive care unit.
(TIF)

S3 Fig Kaplan-Meier curves for TTR in patients receiving different treatments. (A) Kaplan-Meier curves for TTR according to IVIG treatment in patients with no lymphopenia; (B) Kaplan-Meier curves for TTR according to corticosteroid treatment in patients with lymphopenia; (C) Kaplan-Meier curves for TTR according to corticosteroid treatment in patients with no lymphopenia; (D) Kaplan-Meier curves for TTR according to lymphocyte count in patients treated with corticosteroids. IVIG, intravenous human γ-immunoglobulin; TTR, time to recover.
(TIF)

S1 Table Multivariate analysis of factors impacting on duration of hospitalization. (DOCX)

S2 Table Comparison of recovery time of lymphocyte between patients treated with methylprednisolone and patients without methylprednisolone treatment. (DOCX)

## Abbreviations

CoV: coronaviruses
MERS: Middle East Respiratory Syndrome
SARS: Severe Acute Respiratory Syndrome
SARS-CoV-2: Severe Acute Respiratory Syndrome coronavirus 2
COVID-19: 2019 coronavirus infections disease
PaO_2_: pressure of oxygen in arterial blood
FiO_2_: fraction of inspire oxygen
ICU: intensive care unit
CT: computed tomography
TTR: the time to recover
LDH: lactate dehydrogenase
CRP: c-reactive protein
AUC: the area under the curve
ROC: receiver-operator curve
CI: confidence interval
IVIG: human γ-immunoglobulin
ACE2: angiotensin-converting enzyme 2
ACEI: angiotensin-converting enzyme inhibitor
ARB: angiotensin II receptor blocker
