# Supplementary figures and images for: Lymphopenia predicted illness severity and recovery in patients with COVID-19: A single-center, retrospective study
Source: PLoS One. 2020 Nov 18;15(11):e0241659. doi: 10.1371/journal.pone.0241659 (PMC7673513; doi:10.1371/journal.pone.0241659)

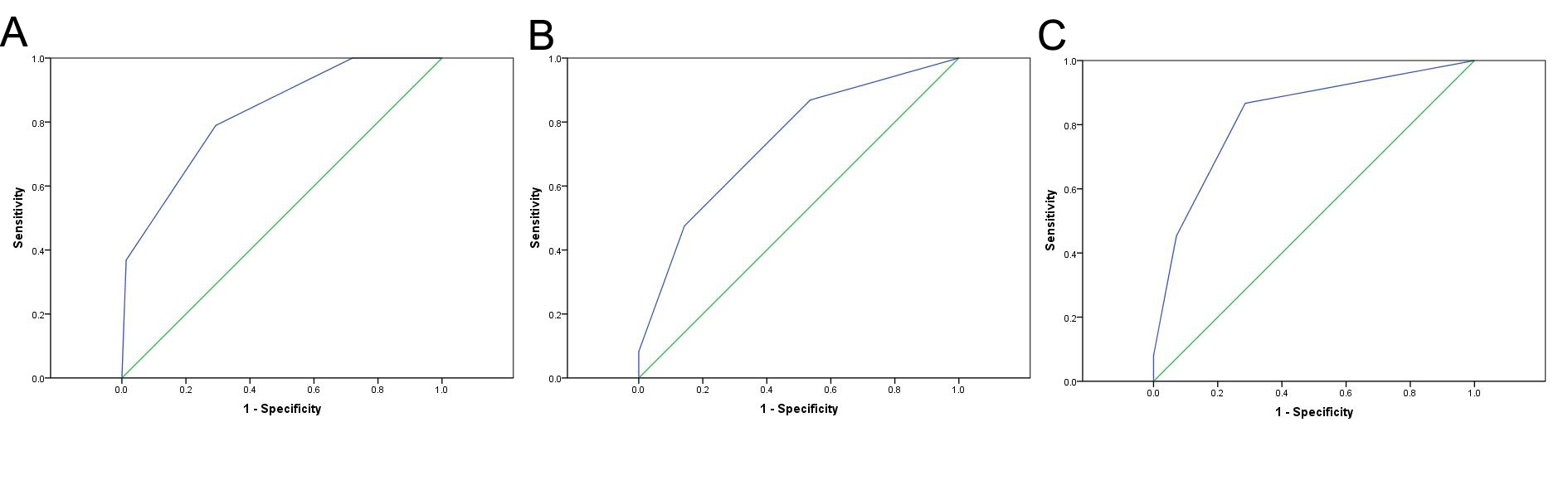

Supplement: S1 Fig — ROC curves to predict patients with (A) pneumonia of severe grade or critical grade, (B) bilateral lung involvement in lung CT scan, (C) abnormal lung image on discharge. The diagonal line indicates an AUC of 0.5 (no discrimination between the two states). CRP, C-reactive protein; LDH, lactate dehydrogenase; AUC, area under the curve; ROC, receiver-operator curve. (TIF) [file pone.0241659.s001.tif]

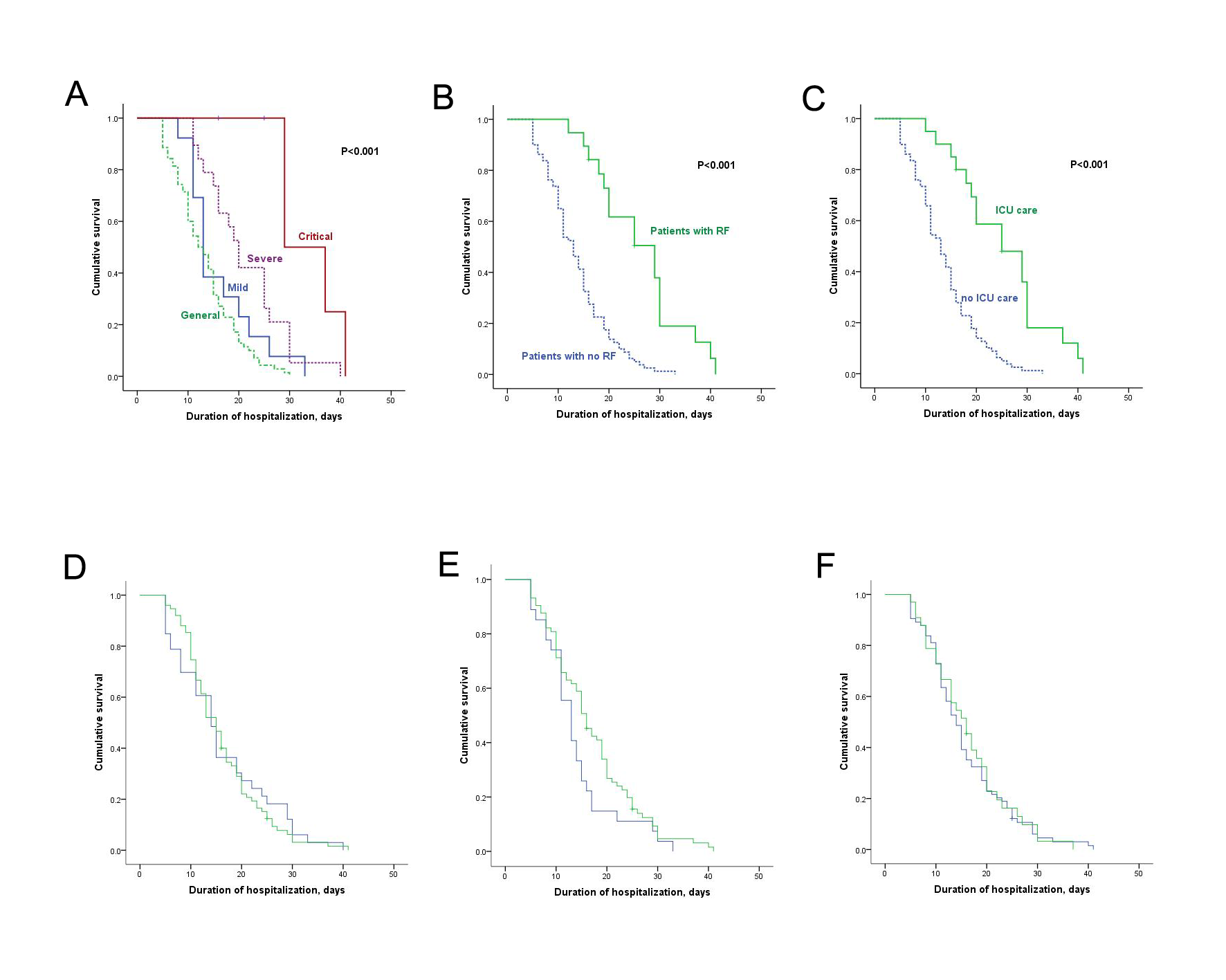

Supplement: S2 Fig — (A) Kaplan-Meier curves for duration of hospitalization according to severity grades of COVID-19; (B) Kaplan-Meier curves for duration of hospitalization according to the presentation of respiratory failure; (C) Kaplan-Meier curves for duration of hospitalization according to the requirement of ICU care; (D) Kaplan-Meier curves for duration of hospitalization according to eosinophil count. The blue line indicates patients with eosinophil counts ≥0.02 × 10⁹/L; The green line indicates patients with eosinophil counts <0.02 × 10⁹/L; P = 0.793; (E) Kaplan-Meier curves for duration of hospitalization according to the CRP levels. The blue line indicates patients with a normal CRP level; The green line indicates patients with an elevated CRP level; P = 0.094. (F) Kaplan-Meier curves for duration of hospitalization according to comorbidity. The blue line indicates patients with no comorbidity; The green line indicates patients with comorbidities; P = 0.782. CRP, C-reactive protein. RF, respiratory failure; ICU, intensive care unit. (TIF) [file pone.0241659.s002.tif]

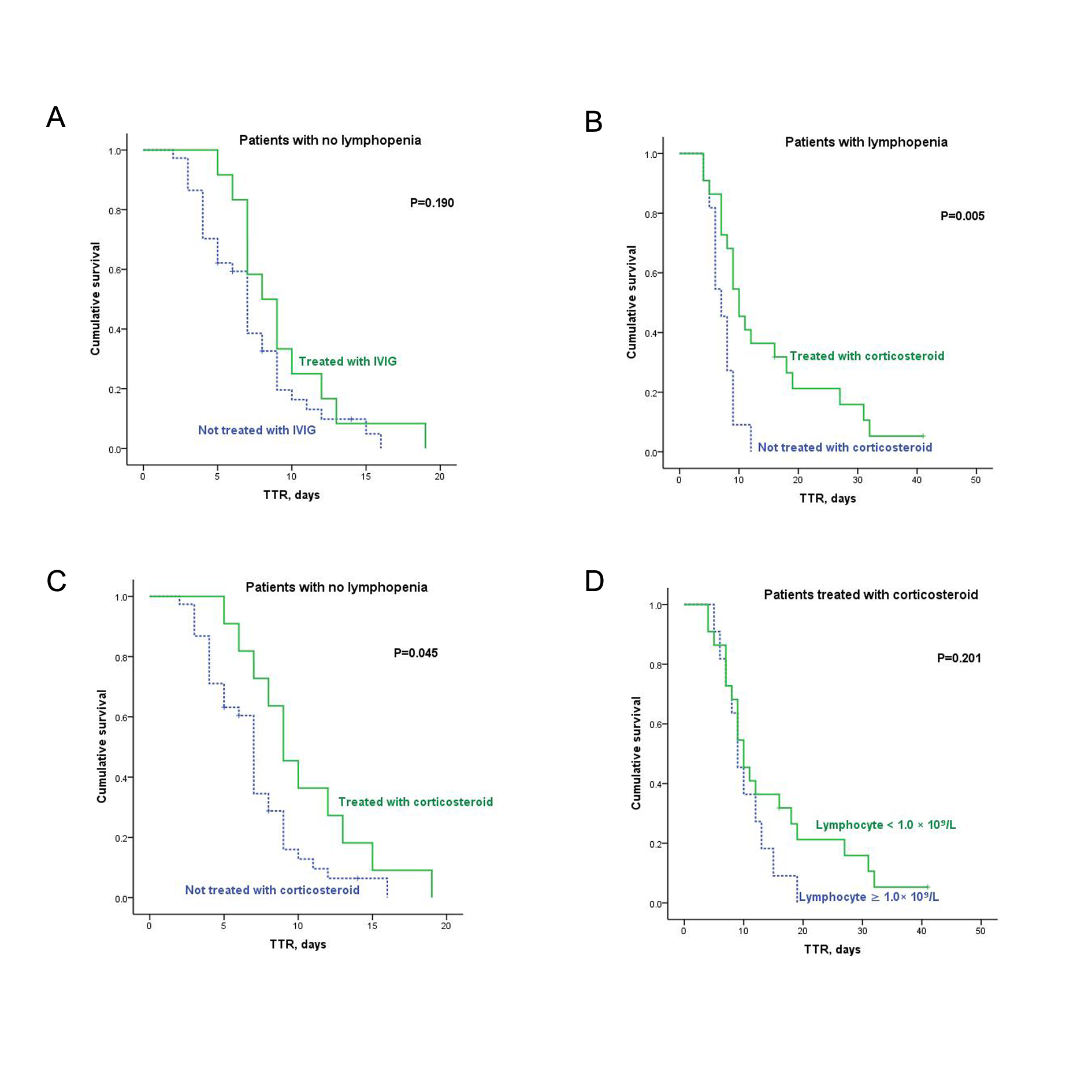

Supplement: S3 Fig — (A) Kaplan-Meier curves for TTR according to IVIG treatment in patients with no lymphopenia; (B) Kaplan-Meier curves for TTR according to corticosteroid treatment in patients with lymphopenia; (C) Kaplan-Meier curves for TTR according to corticosteroid treatment in patients with no lymphopenia; (D) Kaplan-Meier curves for TTR according to lymphocyte count in patients treated with corticosteroids. IVIG, intravenous human γ-immunoglobulin; TTR, time to recover. (TIF) [file pone.0241659.s003.tif]
