# Supplementary material for: Lymphopenia predicted illness severity and recovery in patients with COVID-19: A single-center, retrospective study
Source: PLoS One. 2020 Nov 18;15(11):e0241659. doi: 10.1371/journal.pone.0241659 (PMC7673513; doi:10.1371/journal.pone.0241659)
Supplement: S1 Table — (DOCX) [file pone.0241659.s004.docx]

S1 Table. Multivariate analysis of factors impacting on duration of hospitalization

| Variables | HR (95% CI) | P value |
| --- | --- | --- |
| Lymphopenia | 1.044 (0.648-1.680) | 0.861 |
| Respiratory failure | 0.125 (0.021-0.735) | 0.021 |
| Critical grade of pneumonia | 1.046 (0.701-1.560) | 0.825 |
| ICU care | 1.744 (0.310-9.802) | 0.528 |

HR=hazard ratio; ICU=intensive care unit.
