# Supplementary material for: Lymphopenia predicted illness severity and recovery in patients with COVID-19: A single-center, retrospective study
Source: PLoS One. 2020 Nov 18;15(11):e0241659. doi: 10.1371/journal.pone.0241659 (PMC7673513; doi:10.1371/journal.pone.0241659)
Supplement: S2 Table — (DOCX) [file pone.0241659.s005.docx]

S2 Table. Comparison of recovery time of lymphocyte between patients treated with methylprednisolone and patients without methylprednisolone treatment.

|  | Time to recover (days) | P value |
| --- | --- | --- |
| Patients treated with methylprednisolone (N=22) | 10.45 | 0.039 |
| patients without methylprednisolone treatment (N=13) | 7.38 |  |
